# The autofluorescence characteristics of bacterial intracellular and extracellular substances during the operation of anammox reactor

**DOI:** 10.1038/srep39289

**Published:** 2017-01-16

**Authors:** Xiaolin Hou, Sitong Liu, Ying Feng

**Affiliations:** 1 Department of Environmental Engineering, Peking University, Beijing 100871, China; 2 The Key Laboratory of Water and Sediment Sciences, Ministry of Education, Beijing 100871, China

## Abstract

Anammox is a cost-effective process to treat nitrogenous wastewater. In this work, excitation–emission matrix (EEM) fluorescence spectroscopy was used to characterize the intracellular and extracellular substances of anammox sludge during reactor operation of 276 days. Four main fluorophores were identified from the intracellular substances. Two main protein-like fluorophores were identified from the extracellular substances. Correlation analysis revealed that intracellular 420 peak and humic-like peak had strong correlation with nitrogen removal rate. The two intracellular protein-like peaks had high correlation with MLVSS and MLVSS growth rate. Correlation analysis between different fluorophores discovered that the two peaks in each of these three groups—two intracellular protein-like peaks, two humic acid-like peaks and the two extracellular protein-like peaks had strong intercorrelation, which gave evidence of their homology. A specific method for fluorescence monitoring of anammox reactor were put forward, which included typical fluorescence indexes and their possible values for different operation phases.

Successful biological wastewater treatment process depends largely on high bacterial activity. Three-dimensional excitation–emission matrix (EEM) fluorescence spectroscopy as a rapid, selective and sensitive technique is very helpful for bacterial metabolism monitoring. The use of fluorescence is based on the fact that all microorganisms contain natural intracellular and extracellular fluorophores, whose concentrations depend on the physiological state of the cells^1^,^2^. In fact, many biomolecules, including proteins, enzymes, coenzymes, pigments and primary or secondary metabolites (e.g., fulvic and humic acids), have been found to exhibit a characteristic fluorescence^3^. Protein is one of the most important fluorophores and it exists in most intracellular and extracellular substances. Protein fluorescence can be used to reflect the content of Chemical Oxygen Demand (COD), Soluble Microbial Product (SMP) of wastewater^4^,^5^ and it were also used to describe the volatile solids reduction in aerobic sludge digestion reactors^6^. Humic-like acid is another significant fluorophore and its fluorescence is reported to associate with substrate utilization of bacteria^7^. The fluorescence of Nicotinamide Adenine Dinucleotide (NADH) is also common in anaerobic wastewater treatment process, and information about the physiological response of microbes towards changing culture conditions was acquired^8^. Besides, in biological wastewater treatment reactors, other fluorophores such as fulic-like acid and lactoflavin may also exist and contribute the total fluorescence signals^9^. In recent years, EEM fluorescence spectroscopy has been successfully used to evaluate the characteristics of microorganisms. Especially the supernatant, intracellular and extracellular fluorophores of activated sludge were systematically studied^10^,^11^,^12^,^13^.

Anaerobic ammonium oxidation (anammox) is a biological process that uses nitrite as the electron acceptor to convert ammonium to nitrogen gas under anoxic conditions^14^. Anammox process for wastewater treatment has several advantages, high efficiency, no need of additional carbon source, decreased oxygen demand and low sludge output etc^15^. The researches about fluorescence of anammox sludge are quite few compared to activated sludge. Kartal *et al*.^16^ reported *Candidatus Brocadia fulgida* (a kind of anammox bacteria) had autofluorescent extracellular polymeric substance which had two excitations (352 and 442 nm) and two emissions (464 and 521 nm) maxima. Ruscalleda *et al*.^7^ detected the fluorescence in the effluent of anammox reactor and pointed out that there were two main components corresponding to protein-like (excitation peaks at <240, 280 and 330 nm, and emission at 346 nm) and humic acid-like substances (excitation peaks at <240, 355 and 420 nm, and emission at 464 nm) respectively. Nevertheless, there still exist some deficiencies in this field. Most of the researches only focused on the kinds and intensity change of fluorescence. The relationship between anammox fluorescence and bacterial metabolism, reactor performance still needs more information. Moreover, in the majority of researches, fluorescence data at only one time point or during a short time (such as one cycle of the reactor operation) was obtained. A system fluorescence monitoring with a long time and high frequency is in great need. It is well known that anammox bacteria grow slow and are extremely sensitive to environmental inhibition. Anammox reactors have a long start-up period and strict conditions for operation. Therefore, it is necessary for anammox reactors to be closely monitored. Aiming at this point, fluorescence monitoring can be a proper strategy.

This work aimed at monitoring the fluorescence characteristics of bacterial intracellular and extracellular substances during the anammox reactor operation. The fluorescence data of 6 kinds of fluorophores (4 kinds of intracellular substances and 2 kinds of extracellular substances) during 276 days was systematically and detailed monitored. To provide a better understanding of the relationship between fluorescence characteristics and bacteria metabolic activity, the result was dealt with correlation analysis. The interrelationships between each of the fluorophores were also brought to light. At last, specific methods for fluorescence monitoring of anammox reactor were put forward. This information would be valuable for clarifying the feasibility of fluorescence monitoring of bioprocess.

## Result

### The operation of the anammox reactor

**Figure 1:**
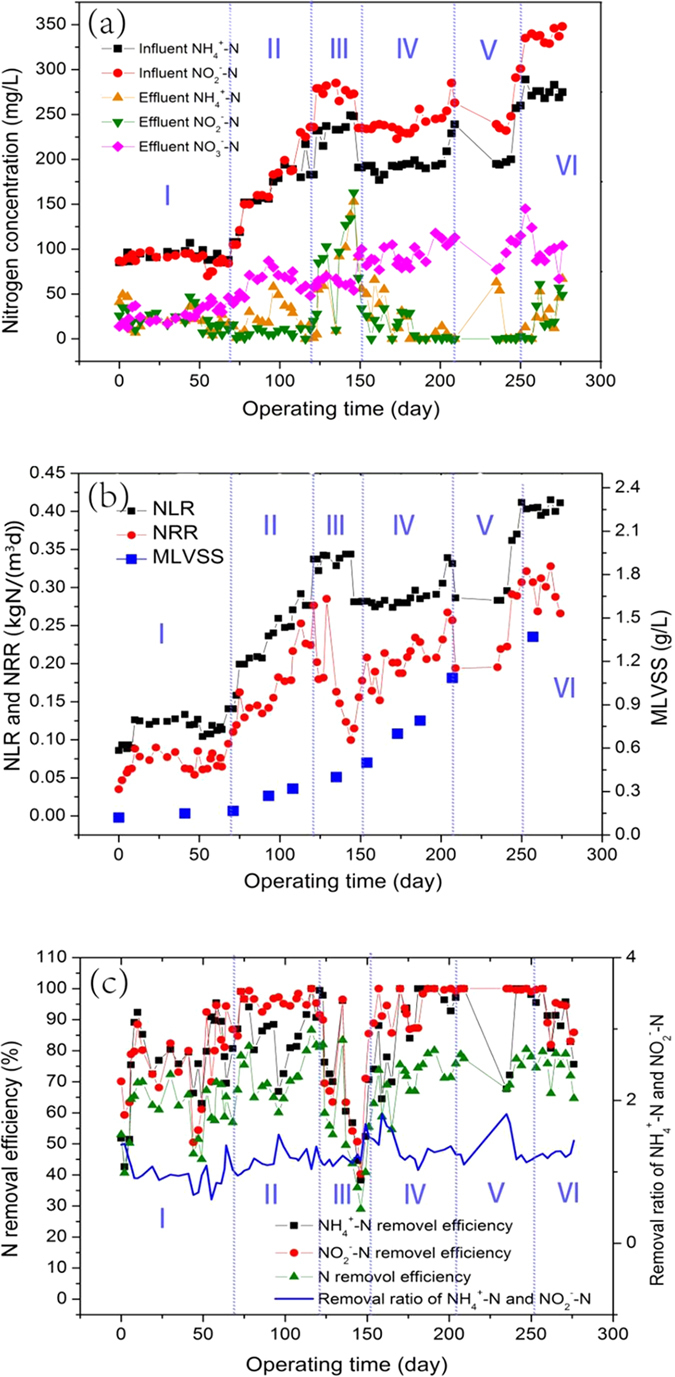
The operation performance of the anammox reactor: (**a**) The Nitrogen concentration of influent and effluent, (**b**) NLR, NRR and MLVSS, (**c**) N removal efficiency, Removal ratio of NH_4_^+^-N and NO_2_^—^N.

The anammox bacteria grow very slowly. MBR has advantage of high sludge retention and therefore 5 L MBR was selected in this study for anammox process operation. The anammox reactor was successfully operated for 276 days. As shown in Fig. 1(a) and (b), the nitrogen removal performance of the reactor was continuously improved during the reactor operation process which could be generally divided into 6 phases as shown in the Material and methods.

First, in phase I, the reactor was run at an influent nitrogen concentration of 100 mg/LNH_4_^+^-N and NO_2_^−^-N. The NH_4_^+^-N and NO_2_^−^-N in the effluent changed from 5 mg/L to 51 mg/L, which indicated the unstability of the reactor in the early start-up process. In phase II, the influent concentration had a great increase along with the improvement of reactor performance. During this period, the NH_4_^+^-N and NO_2_^−^-N concentration in the effluent was about 20 mg/L and 30 mg/L respectively. Unfortunately, due to the over quick increase of influent concentration in the phase II, the reactor showed a inhibition from the 120^th^ day when the NH_4_^+^-N and NO_2_^−^-N concentration in the influent reached 230 mg/L and 280 mg/L. The NH_4_^+^-N and NO_2_^−^-N concentration in the effluent quickly increased to about 150 mg/L in phase III. In order to recover the activity of bacteria, the influent nitrogen concentration of NH_4_^+^-N and NO_2_^−^-N was decreased in phase IV. At the end of this phase, effluent nitrogen concentration of NH_4_^+^-N and NO_2_^−^-N was recovered to around 20 mg/L. From the 210^th^ day, the reactor was suspended and the bacteria were stored in the fridge for 24 days (phase V). The reactor was restarted up at the 234^th^ day. A rapid rise of activity was shown afterwards. In phase VI, the influent nitrogen concentration of NH_4_^+^-N and NO_2_^−^-N reached to 280 and 350 mg/L. At the same time, the effluent NH_4_^+^-N and NO_2_^−^-N concentration was around 20 to 50 mg/L, which demonstrated excellent performance of the reactor.

The NRR, NLR and MLVSS were shown in Fig. 1(b). NRR was around 0.75 kgN/(m^3^d) in the phase I and then quickly increased to 0.27 kgN/(m^3^d) in phase II. NRR had an obvious decrease to 0.10 kgN/(m^3^d) in phase III due to the inhibition of bacteria. Afterwards, it was gradually recovered to 0.28 kgN/(m^3^d) in phase IV. After a short time of suspension (phase V), the NLR and NRR reached 0.42 and 0.33 kgN/(m^3^d) at the end of the operation (phase VI). The MLVSS increased with the start-up period. At the end of the operation, the MLVSS was 1.36 g/L, almost 14 times of its initial value.

- The N removal efficiency of NH_4_^+^-N and NO_2_^−^-N was also calculated. As shown in Fig. 1(c), the TN removal efficiency was kept at around 70%. Only in the lag phase or the inhibition phase, the value dropped to 40% and 28%, respectively. In the end of the operation, the TN removal efficiency reached around 80%. The removal efficiency of NO_2_^−^-N and NH_4_^+^-N undulated greatly during the whole operation, but in the most of the time they were above 75%. In most case, the removal ratio of NO_2_^−^-N and NH_4_^+^-N was kept about 1.32.

### Fluorescent characteristics of intracellular and extracellular substances from anammox sludge

**Figure 2:**
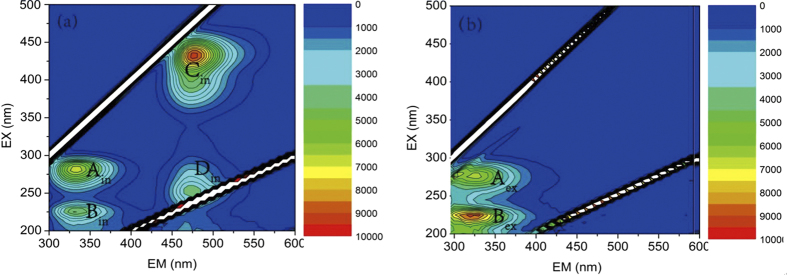
The EEM spectra of (**a**) intracellular and (**b**) extracellular substances.

EEM spectrum was used in this study to analyze the fluorescent characteristics of intracellular and extracellular substances. The result is exhibited in Fig. 2. In the spectra of intracellular substances (Fig. 2(a)), there were four peaks corresponding to four kinds of substances. The first main peak was identified at excitation/emission wavelengths (Ex/Em) of 225/342 nm (Peak A_in_), while the second main peak was identified at Ex/Em of 280/347 nm (Peak B_in_). The two peaks have been described as protein-like peaks, in which the fluorescence is associated with the aromatic (including tryptophanand tyrosine) protein-like substances and tryptophan protein-like substances respectively (Baker, 2001; Chen *et al*.^17^). Another two peaks were located around Ex/Em = 420/470 nm (PeakC_in_) and Ex/Em = 250/470 nm (Peak D_in_). A similar fluorescence signal has also been observed for humic acid-like fluorescence^17^,^18^. In particular, because Peak C_in_ had special significance in this study, it was named “420 peak”.

For the extracellular substances of anammox sludge (Fig. 2(b)), there were two fluorescent peaks which had the same location as Peak A_in_ and Peak B_in_. The first peak at Ex/Em of 280/330 nm (Peak A_ex_) is corresponding to tryptophan protein-like substances, while the second peak located around Ex/Em = 225/330 nm (Peak B_ex_) was identified to be the aromatic protein-like peak^17^,^19^.

Through the monitoring of EEM, the fluorescence characteristics of intracellular and extracellular substances were detailedly recorded. For the four intracellular fluorescent peaks, their intensity generally increased with operation of the reactor. To go further, two protein-like peaks (Peak A_in_ and Peak B_in_) had the similar variation trend, and they have frequent fluctuation during the whole operation. The 420 peak and the humic acid-like peak (Peak C_in_ and Peak D_in_) had the similar tendency. Their intensity stably increased and had small fluctuation. Three times of decrease for the peak intensity of Peak C_in_ and Peak D_in_ (the 188^th^ day, the 224^th^ day and the 260^th^ day) was corresponding to three times of NRR drop. By comparison, the intensity of extracellular fluorescent peaks (Peak A_ex_and Peak B_ex_) had greater fluctuation than Peak A_in_ and Peak B_in_ during the operation. Peak A_ex_ and Peak B_ex_ also had the similar tendency.

**Figure 3:**
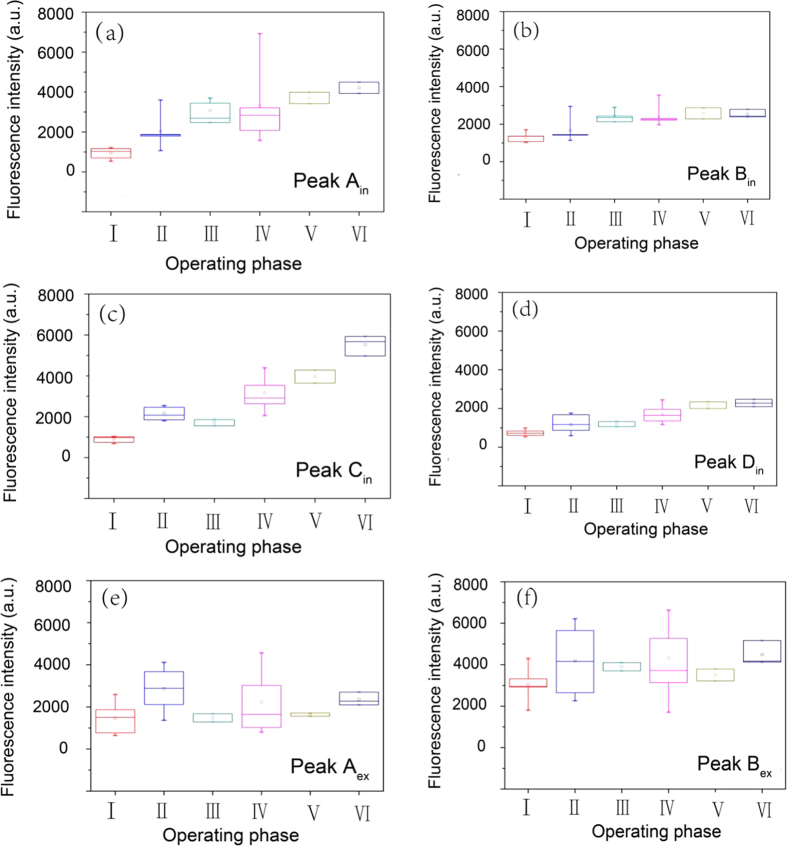
The fluorescence intensity variation of (**a**) Peak A_in_: intracellular tryptophan protein-like substances, (**b**) Peak B_in_: intracellular aromatic protein-like substances, (**c**) Peak C_in_: intracellular 420 substances, (**d**) Peak D_in_: intracellular humic-like substances, (**e**) Peak A_ex_: extracellular tryptophan protein-like substances, (**f**) Peak B_ex_ extracellular aromatic protein-like substances in different operation phases.

In order to go deeper into the variation of fluorescence, box plots were used to show the fluorescence characteristics in different start-up phases of reactor. The results were shown in Fig. 3: (i) The intensity of Peak A_in_ and Peak B_in_ has similar trend and stably increased with the operation phase (Fig. 3(a) and (b)). (ii) The intensity of Peak C_in_ and Peak D_in_ had a more remarkable change (Fig. 3(c) and (d)). Their intensity increased distinctly in Phase II, during which the reactor had a rapid performance improvement. Afterwards, the intensity dropped in the Phase III in which the bacteria are inhibited by the high-concentration influent. However, the variation range of Peak D_in_ was much smaller than Peak C_in_. (iii) For extracellular substances, the intensity of Peak A_ex_ and Peak B_ex_ had a relatively lager range of variation than intracellular ones (Fig. 3(e) and (f)). What’s more, in the Phase II, IV and VI when the performance of reactor was relatively high, the intensity of extracellular protein-like peaks also had a high value, which meant more EPS production. While in the Phase III and V the bacteria were in inhabitation or the reactor was suspended, the intensity of Peak A_ex_ and Peak B_ex_ dropped, which indicated the degradation of EPS.

### The relationship between fluorescence characteristics and sludge properties

**Figure 4:**
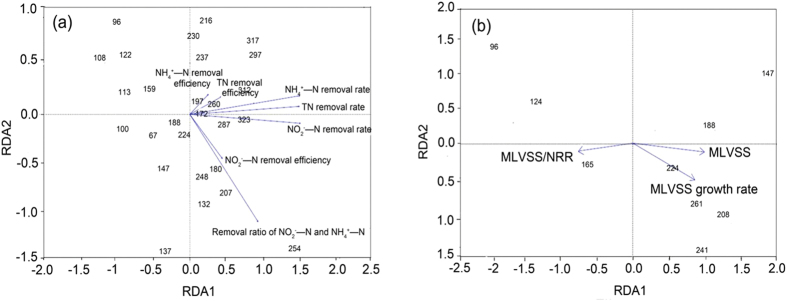
The impact of sludge properties on fluorescence characteristics (RDA).

In order to reveal the relationship between the sludge properties and fluorescence characteristics, RDA method was utilized as shown in Fig. 4. On account of less data of MLVSS than other property indexes, an independent figure was prepared for three values, MLVSS, growth rate and NRR/MLVSS. In Fig. 4, the length of arrows demonstrated how strong the impact of these sludge properties on the fluorescence characteristics. It can be seen from Fig. 4(a) that the removal rate of TN, NH_4_^+^-N and NO_2_^−^-N, the removal ratio of NH_4_^+^-N and NO_2_^−^-N were the indexes highly affect the fluorescence intensity. Furthermore, the impact of MLVSS and MLVSS growth rate were higher than that of NRR/MLVSS (Fig. 4(b)).

In order to obtain specific correlation coefficient of fluorescence intensity and sludge properties indexes, Statistical Product and Service Solutions (SPSS) software was employed to do correlation analysis. The results were shown in Table 1. The sludge properties contained 10 indexes exhibited in the first column on the left. The fluorescence characteristics shown in the first row consisted of 6 kinds of fluorescence in the extracellular and intracellular substances.

First, the data in the Table 1 marked with three asterisks stand for very strong correlation (the R value was from 0.8 to 1.0). It can be seen that both intensity of 420 peak and intracellular humic acid-like peak (Peak C_in_ and Peak D_in_) had a strong correlation with N removal rate (TN, NH_4_^+^-N and NO_2_^−^-N). The R value for Peak C_in_ was 0.896, 0.879, 0.877 respectively, while that for the intensity of Peak D_in_ were 0.849, 0.866, 0.823, which were a little lower. This means by comparison, Peak C_in_ have an excellent ability in the reflection of sludge activity and N removal rate. Besides, the intracellular tryptophan and aromatic protein-like substances (Peak A_in_ and Peak B_in_) had strong correlation with MLVSS. The Peak A_in_ also had strong correlation with MLVSS growth rate. This result indicates that intracellular protein-like substances can reflect the growth of sludge. Second, the data marked with two asterisks were attributed to groups with medium correlation (the value R was from 0.6 to 0.8). It can be seen that two kinds of intracellular protein-like substances (Peak A_in_ and Peak B_in_) also had a passable correlation with NRR. Moreover, Peak C_in_(0.693) and Peak D_in_(0.650) had medium correlation with MLVSS. Third, the data marked with one asterisk stand for very weak correlation (the value R was from 0.4 to 0.6). The correlation coefficient between the removal ratio of NH_4_^+^-N and NO_2_^−^-N and the intensity of each fluorescence peak (except Peak D_in_) was all in the range of 0.45 to 0.50. The intracellular aromatic protein-like substances had a correlation coefficient of 0.501 with MLVSS growth rate. Other data indicates the R value is lower than 0.4, which means no obvious correlation.

### The relationship between different fluorescent substances

In order to describe the relationship between different fluorescent substances, correlation analysis was also conducted on each two fluorescent substances. The results are shown in Table 2. The group with the highest correlation was two intracellular protein-like substances (Peak A_in_ and Peak B_in_) (marked with two asterisks) and their R value was as high as 0.836. Furthermore, intracellular 420 substances (Peak C_in_) and humic acid-like substances (Peak D_in_)(0.668), two extracellular protein-like substances (Peak A_ex_ and Peak B_ex_)(0.702) also had superior correlation (marked with one asterisk). This kind of high correlation demonstrates the homology of each group.

**Figure 5:**
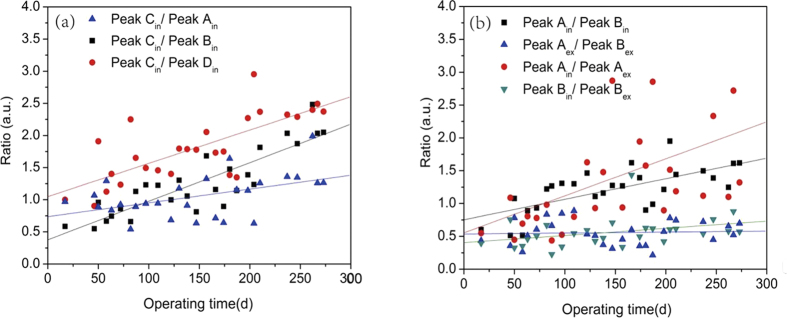
The intensity ratio of different fluorescent peaks: (**a**) the intensity ratio of intracellular420 peak (Peak C_in_) to another three kinds of intracellular substances, (**b**) the intensity ratio of intracellular and extracellular protein-like substances.

To go further, ratio between each two fluorescent peaks was used to indicate relative variation of different fluorophores. The change of ratio between each two fluorescent peaks with operation time was exhibited in Fig. 5. Figure 5(a) showed the ratios between 420 substances and other three intracellular fluorescent substances. It can be seen that these ratios continuously increased with the time, indicating a higher producing rate of 420 substances^7^. Figure 5(b) revealed the relationship between different protein-like substances. The ratio of intracellular to extracellular tryptophan protein-like substances the most rapidly increased with the time but with great fluctuation. The ratio of intracellular tryptophan to aromatic protein-like substances took the second place. The ratio of two extracellular protein-like substances and the ratio of intracellular to extracellular aromatic protein-like substances are relatively stable during the whole start-up period.

## Discussion

This study detailedly described the change profile of EEM fluorescence characteristics in the different phases during reactor operation of 276 days. In fact, adjusting HRT will result in the varied influent loading rates, the effects of which on the EEM fluorescence characteristics have been investigated (Figs 1 and 3).

During the 276 days’ reactor operation, the influent pH and reactor temperature were maintained at 7.3 ± 0.1 and 37 ± 1 °C to probe optimal anammox metabolism condition and frequently used anammox reactor parameters, with aim to explore the fluorescence characteristics variety of anammox reactor operation. Therefore, we did not investigate the effects of different temperatures on EEM characteristics. With the improvement of reactor performance, the intracellular and extracellular fluorescent substances showed different characteristics in different operation phases. This is in coincidence with many other researches^5^,^7^,^9^,^20^. Results indicated EEM fluorescence has high relativities with reactor NH_4_^+^, NO_2_^−^, TN removal performance (Fig. 4). Therefore, EEM fluorescence characteristics could be applied for monitoring reactor performance (NH_4_^+^ removal, NO_2_^−^ removal and TN removal performance). Anammox activity has also been measured according to the nitrogen removal rate and biomass in the reactor operation process (NRR/MLVSS). It did not present obvious correlation with the EEM fluorescence characteristics (Fig. 4). It could be understood that reactor performance could not be well reflected by anammox activity, and the growth of MLVSS also contributed to the varied reactor performance. This consists with the results show in Fig. 4. Extracellular fluorescence of aerobic and anaerobic sludge has been reported in the previous study. The protein-like peaks including Ex/Em = 225/342, Ex/Em = 280/347 were usually found with higher intensity of the former one^2^, the location and tendency of which was in accordance with this study. In fact, the peak location sometimes varied in different biological samples. For example, Zhu *et al*. found the protein-like peak located at Ex/Em = 220–230/358–364 and Ex/Em = 280–290/360–368^11^. For the humic acid-like peaks, the location is totally different in the reports, including Ex/Em = 330/420 and Ex/Em = 235–240/417–456^2^,^10^,^11^. In this study, the humic acid-like peak with high intensity emerged at Ex/Em = 420/470 and Ex/Em = 250/470. Interestingly, the fluorescence with Ex/Em = 420/470 peak has an identical location with coenzyme F420 which is reported unique in the methanogens bacteria. However, the existence of methanogens was denied by the negative PCR results. Therefore, its existence and good relationship with reactor performance indicated the some related substances may be a kind of coenzyme corresponding to substrate metabolism in this reactor.

To get further information, we found intracellular 420 substances had a higher correlation and larger variation range compared to humic-like substances. In particular, this is the first report of 420 peak (Ex/Em = 420/470) in anammox sludge. In many studies, this peak was used to reflect the methane production activity, because coenzyme F420 plays a significant role in heterotrophic metabolism pathway^21^,^22^. However, when Polymerase Chain Reaction for functional gene mcrAr of methanogenst was applied to find the existence of methanogens, we did not get the positive results. Ruscalleda *et al*.^7^ and Elliott *et al*.^23^ pointed out that humic acid-like fluorescence had close connection with substrate utilization, which is in coincidence with our work.

Two kinds of intracellular protein-like substances had high correlation with MLVSS and MLVSS growth rate. This is in agreement with the previous result^9^. With the growth of anammox bacteria, they tend to have lager density in mixed liquor of the reactor. When put in the mould for EEM measurement, they become a more compact layer. Besides, the protein inside the cell is also fast produced. This leads to the increase of intracellular protein-like fluorescence intensity. More MLVSS can in turn lead to larger MLVSS growth rate. Therefore, intracellular protein-like fluorescence (especially tryptophan protein-like fluorescence) can be used as an index to indicate the sludge growth.

Moreover, for extracellular protein-like substances, though they did not have obvious correlation with any sludge properties due to their fluctuation, there was still a variation rule of them. When performance of the reactor was relatively high in phase II and VI, the intensity of extracellular protein-like peaks had an apparent increase, which meant more EPS production. However, when the reactor was in inhabitation or suspended, the intensity of these two peaks dropped, which indicated the degradation of EPS. Research showed EPS can be used as nutrition for metabolism, which explains the variation of extracellular protein-like substances with reactor performance.

According to Li *et al*.^9^, intracellular 420 substances, humic acid-like substances and extracellular protein-like substances are called utilization associated products (UAP), which are associated with substrate utilization. In contrast, the two intracellular protein-like substances are biomass-associated products (BAP), which are associated with biomass growth. For the relationship between different fluorescent substances, three groups with high correlation were picked out. They were two intracellular protein-like substances, two extracellular protein-like substances, intracellular 420 substances and humic acid-like substances respectively. High correlation gave evidence of the homology for each group. They may compose cell structure together in a certain proportion. The intensity ratio of intracellular 420 peak to other three intracellular peaks increased with the operation time. This indicates that, along with the reactor performance improvement, intracellular 420 substances had faster production rate than other three kinds of substances, which supported the surmise that 420 substances may be a kind of coenzyme corresponding to substrate metabolism.

For the relationship between different protein-like substances, it was reported that, in the hydrolytic process of sludge, the ratio of tryptophan to aromatic protein-like substances in EPS had a notable decrease^12^. In contrast, this value increased during the sludge granulation^11^. In our study, though there were no data of aggregation ability, it could be observed that the sludge granules became bigger and settled faster during the operation process. For fluorescence characteristics, it is illustrated that the ratio of intracellular tryptophan protein-like substances to intracellular aromatic protein-like substances increased substantially. But that of the two extracellular protein-like substances did not obviously change, which is different from previous researches. For its reason, we suppose the great fluctuation of EPS fluorescence was an important influence factor. That means the EPS of bacteria was produced fast and also degraded fast, which led to disordered variation of the ratio of two extracellular protein-like substances during the operation process. By contrast, intracellular protein-like substances had relatively stable amounts and they also had frequent exchange with EPS at the same time. For anammox bacteria, frequent variation of EPS amounts may be a unique characteristic.

Based on all of the analysis above, a specific method for monitoring the reactor performance for this reactor was put forwarded as shown in Table 3. EEM fluorescence characteristics and reactor performance data in the five different operational phases were collected and analyzed to get a specific method for monitoring the reactor performance for this reactor. Different characteristic parameters were used for different reactor performance phases. For the lag phase, intracellular 420 peak (Peak C_in_) and humic-like peak (Peak D_in_) were good indexes. Their values were both lower than 1500. The ratios of Peak A_in_/Peak B_in_ (<1.0) and Peak C_in_/Peak D_in_ (<1.5) were also typical indexes for this phase. Accordingly, for the high reactor performance phase, intracellular 420 peak (Peak C_in_) and humic-like peak (Peak D_in_) were higher than 5000 and 2100, respectively. The ratios of Peak A_in_/Peak B_in_and Peak C_in_/Peak D_in_were higher than 2.3 and 1.5, respectively. Furthermore, when the performance of the reactor was in improvement, the intensity of intracellular 420 peak (Peak C_in_) and humic-like peak (Peak D_in_) was both increasing. The extracellular tryptophan protein-like peak (Peak A_ex_) and aromatic protein-like peak (Peak B_ex_) also increased because of more production of EPS under this circumstance. Accordingly, when the bacteria were in inhibition, the intensity of Peak C_in_, Peak D_in_, Peak A_ex_ and Peak B_ex_ all decreased. For the high bacterial growth phase, the intracellular tryptophan protein-like peak (Peak A_in_) and intracellular aromatic protein-like peak (Peak B_in_) were good indexes. Their intensity was higher than 4000 and 2500 respectively, which indicated the bacteria were in high growth rate. Besides, in this situation the intensity of Peak A_ex_ and Peak B_ex_ was higher than 2200, 4200 respectively due to the fast accumulation of EPS.

Correlation analysis revealed that intracellular 420 peak and humic-like peak had strong correlation with nitrogen removal rate. The two intracellular protein-like peaks had high correlation with MLVSS and MLVSS growth rate. Intracellular tryptophan protein-like substances had higher producing rate than extracellular tryptophan and intracellular aromatic protein-like substances, which possibly related to bacterial aggregation. The typical fluorescence indexes and their values in different operation phases indicate the potential method for fluorescence monitoring of anammox reactor.

## Methods

### Membrane Bioreactor operation

5 L Membrane Bioreactor (MBR) was fed with a synthetic high-nitrogen wastewater. Seeding sludge was taken from sequencing batch reactors operated in our laboratory and it was consist of *Candidatus Brocadia fulgida*^24^. The reactor’s Hydraulic Residence Time (HRT) was maintained at 1.5 day during the whole operation. Temperature was maintained at 37 ± 1 °C by a water bath. The reactor was continuously filled from the bottom with mineral medium prepared with distilled water. The concentrated solutions of substrates and trace elements adapted from van de Graaf *et al*.^25^. To ensure anoxic conditions, the reactor was sealed and the influent was sparged for 30 min with N_2_/CO_2_ (50:1) gas.

The reactor was operated for six phases over a time period of 276 days. They were lag phase (I, day 0- day 70), performance improvement phase (II, day 70- day 120), inhibition phase (III, day 120- day 150), recover phase (IV, day150- day 210), suspension phase (V, day 210- day 250) and high performance phase (VI, day 250- day 276), respectively. The initial influent concentration of NH_4_^+^-N and NO_2_^−^-N was around 100 mg/L in the phase I. In phase II, the influent nitrogen concentration had a great improvement (280 mg/L NO_2_^−^-N and 230 mg/L NH_4_^+^-N at the 120^th^ day). The ratio of NO_2_^−^-N and NH_4_^+^-N was kept at about 1.3 (corresponding to the anammox stoichiometric characters) in the influent during the following operation stage. Unfortunately, due to the over quick increase of influent load, the bacteria were inhibited during the 120^th^ day to 150^th^ day in the phase III when the influent concentration of NH_4_^+^-N and NO_2_^−^-N was around 230 and 280 mg/L, respectively. In order to recover the performance of the reactor, the influent nitrogen concentration of NH_4_^+^-N and NO_2_^−^-N was decreased to 180 and 230 mg/L in the phase IV. Then in the phase V, the reactor was suspended for 24 days. The bacteria were taken out and kept at −20 °C. The reactor was restarted up on the 234^th^ day. The nitrogen concentration for restart-up was set at 200 mg/L and 250 mg/L for NH_4_^+^-N and NO_2_^−^-N, respectively. In the phase VI, the corresponding nitrogen concentration of NH_4_^+^-N and NO_2_^−^-N reached to 280 and 350 mg/L. The reactor showed relatively high and stable performance.

The influent and effluent samples were collected on daily basis and analyzed immediately. The determination of pH, ammonium, nitrite, nitrate and volatile suspended solids (VSS) concentrations were carried out following the Standard Methods^26^. The VSS was measured every ten days and other parameters were measured every three days.

### Extraction of extracellular and intracellular substances

Extracellular polysaccharide (EPS) was extracted using the cation exchange resin (CER) method^27^. 10 mL mixing sludge solution from the reactor was harvested. Then bacterial pellets were obtained using centrifugation at 4000 g for 10 min, which were washed three times with 0.1 M NaCl solution. The bacterial pellet was resuspended to a predetermined volume, and CER was added at a dosage of 70 g/g VSS. The suspensions were stirred for 3 h at 200 rpm and 4 °C. Afterwards, the suspensions were settled for 3 min to remove the CER. EPS were harvested after centrifugation at 9000 g and 4 °C for 20 min. The supernatants were filtered through 0.45 μm acetate cellulose membranes (Advantec Co., Japan) to obtain EPS solution for EEM fluorescence spectra. Meanwhile, the pellet was also collected and put in a special mould made of quartz. The bacterial pellet was refashioned to a uniform thin layer with smooth surface in the mould. This mould can be directly used for EEM scan to acquire intracellular fluorescence data.

### EEM fluorescence spectra

All EEM spectra were measured using a luminescence spectrometry (LS-55, Perkin-Elmer Co., USA). The extracellular and intracellular EEM spectra were collected with subsequent scanning emission spectra from 300 to 600 nm at 3 nm increments by varying the excitation wavelength from 200 to 500 nm at 3 nm increments. Excitation and emission slits were both maintained at 10 nm, and the scanning speed was set at 1200 nm/min for all measurements.

UV/Vis absorbance spectra (SPECTROCo., Germany) were used to measure the absorbance of the samples. Results indicated that the excitation wavelengths of the EEM fluorescence spectra exceeded 200 nm, where the absorbance of the samples was lower than 0.1 (Fig. 4). Therefore the inner filter effect can be neglected. The software MatLab 7.0 (MathWorks Co., USA) was employed for handling EEM data. The X-axis represents the emission spectra from 300 to 600 nm, while the Y-axis is the excitation spectra from 200 to 500 nm.

### EEM fluorescence data modeling

In this work, the parallel factor (PARAFAC) analysis was used to model the EEM fluorescence data. They were decomposed using the PARAFAC approach into a structured part where the information is contained, and into a noise part. For each component in the structural part, one score vector and two loading vectors, i.e., emission and excitation, are generated. Before analysis, the raw EEM fluorescence spectra were obtained. As light scatter effects can form diagonal lines in the landscapes and it does not follow the tri-linear structure required for the PARAFAC to work, the EEM data close to the Rayleigh scattering line were set as zero to eliminate the interfere of the Rayleigh scattering on the PARAFAC analysis.

### Statistical Analysis

SPSS (Statistical Product and Service Solutions) software (version 19.0, IBM Co., USA) was employed to do correlation analysis. The correlation coefficient of fluorescence intensity and sludge property indexes was calculated. The correlation coefficient of each two kinds of fluorescence was also figured out. Multivariate analysis of fluorescence intensity and sludge property profiles was done using CANOCO software (Microcomputer Power Co., USA). RDA (redundancy analysis) was used to test the influence of sludge property factors on the variation of fluorescence data. RDA is a constrained ordination technique, based on PCA (Principal Components Analysis), in which ordination axes are constrained to be linear combinations of environmental variables. This technique helps assess the relationship between environmental variables and the multivariate data.

## Additional Information

**How to cite this article**: Hou, X. *et al*. The autofluorescence characteristics of bacterial intracellular and extracellular substances during the operation of anammox reactor. *Sci. Rep.* **7**, 39289; doi: 10.1038/srep39289 (2017).

**Publisher's note:** Springer Nature remains neutral with regard to jurisdictional claims in published maps and institutional affiliations.

## Figures and Tables

**Table 1:** The correlation analysis of fluorescence characteristics and sludge properties.

	Peak A_in_	Peak B_in_	Peak C_in_	Peak D_in_	Peak A_ex_	Peak B_ex_
NRR	0.678**	0.648**	0.896***	0.849***	0.221	0.218
NH_4_^+^-N removal rate	0.696**	0.680**	0.879***	0.866***	0.168	0.177
NO_2_^−^-N removal rate	0.696**	0.683**	0.877***	0.823***	0.257	0.271
N removal efficiency	0.109	0.022	0.309	0.255	0.126	-0.075
NH_4_^+^-N removal efficiency	0.072	0.019	0.177	0.179	0.072	0.014
NO_2_^−^-N removal efficiency	0.258	0.092	0.274	0.151	0.316	0.082
Removal ratio of NH_4_^+^-N and NO_2_^−^-N	0.454*	0.449*	0.469*	0.369*	0.478*	0.496*
MLVSS	0.827***	0.813***	0.693**	0.650**	0.133	0.308
MLVSS growth rate	0.805***	0.501*	0.367	0.371	0.164	0.162

**Table 2:** The correlation analysis of different fluorescent substances.

	Peak A_in_	Peak B_in_	Peak C_in_	Peak D_in_	Peak A_ex_	Peak B_ex_
Peak A_in_	1.000	0.836**	0.314	−0.134	0.327	0.215
Peak B_in_	0.836**	1.000	0.342	.0.155	0.124	0.264
Peak C_in_	0.314	0.342	1.000	0.668*	−0.014	0.033
Peak D_in_	−0.134	0.155	0.668*	1.000	−0.428	−0.119
Peak A_ex_	0.327	0.124.	−0.014	−0.428	1.000	0.702*
Peak B_ex_	0.215	0.264	0.033	−0.119	0.702*	1.000

**Table 3:** Specific fluorescence parameters for monitoring the anammox reactorperformance at different operation phase.

	Peak A_in_	Peak B_in_	Peak C_in_	Peak D_in_	Peak A_ex_	Peak B_ex_	Peak A_in_/Peak B_in_	Peak C_in_/Peak D_in_
Lag Phase	−	−	<1500	<1500	−	−	<1.0	<1.5
Performance improvement phase	−	−	Intensity increase	Intensity increase	Intensity increase	Intensity increase	−	−
Inhibition phase	−	−	Intensity increase	Intensity increase	Intensity increase	Intensity increase	−	−
High Performance phase	−	−	5000−5900	2100−2500	−	−	>1.5	>2.3
High bacterial growth phase	>4000	>2500	−	−	>2200	>4200	−	−
